# Fabrication of methylene blue-loaded ovalbumin/polypyrrole nanoparticles for enhanced phototherapy-triggered antitumour immune activation

**DOI:** 10.1186/s12951-022-01507-5

**Published:** 2022-06-22

**Authors:** Xiao Xu, Huafen Mao, Yunchao Wu, Suwan Liu, Jingjin Liu, Qianzhe Li, Mengyu Yang, Jinqian Zhu, Shengqiang Zou, Fengyi Du

**Affiliations:** 1 Affiliated Third Hospital of Zhenjiang, Jiangsu University, Zhenjiang, 212013 People’s Republic of China; 2 School of Medicine, Jiangsu University, Zhenjiang, 212013 People’s Republic of China; 3 Lianyungang Maternal and Child Health Hospital, Lianyungang, 222000 People’s Republic of China; 4 Clinical Laboratory, The Third People’s Hospital of Changzhou, Changzhou, 213001 People’s Republic of China

**Keywords:** Polypyrrole nanoparticles, Methylene blue, Laser therapy, Immunotherapy

## Abstract

**Background:**

Phototherapy-triggered immunogenic cell death (ICD) rarely elicits a robust antitumour immune response, partially due to low antigen exposure and inefficient antigen presentation. To address these issues, we developed novel methylene blue-loaded ovalbumin/polypyrrole nanoparticles (MB@OVA/PPY NPs) via oxidative polymerization and π–π stacking interactions.

**Results:**

The as-prepared MB@OVA/PPY NPs with outstanding photothermal conversion efficiency (38%) and photodynamic properties were readily internalized into the cytoplasm and accumulated in the lysosomes and mitochondria. Upon 808 nm and 660 nm laser irradiation, the MB@OVA/PPY NPs not only ablated tumour cells by inducing local hyperthermia but also damaged residual tumour cells by generating a large amount of reactive oxygen species (ROS), finally triggering the release of many damage-associated molecular patterns (DAMPs). Moreover, the MB@OVA/PPY NPs synergized with DAMPs to promote the maturation and improve the antigen presentation ability of DCs in vitro and in vivo.

**Conclusions:**

This work reported a PPY NPs-based nanoplatform to encapsulate the therepeutic proteins and absorb the functional molecules for combination therapy of tumours. The results demonstrated that the prepared MB@OVA/PPY NPs could be used as effective nanotherapeutic agents to eliminate solid tumours and trigger a powerful antitumour immune response.

## Introduction

Phototherapy has received extensive attention in tumour treatment over the past few years, as it provides greater safety and effectiveness than traditional therapies [1, 2]. A great deal of research and effort has been devoted to developing noninvasive, targeted and highly effective treatments against tumours [3, 4]. For instance, photothermal therapy (PTT), as an effective laser-based therapy, can convert near infrared light energy into heat to kill tumour cells based on the accumulation of photosensitizers in the tumour [5, 6]. Compared to traditional chemotherapy, radiotherapy, surgery and treatments, PTT has strong, noninvasive, effective targeting characteristics and does not cause severe side effects like traditional treatments [7, 8]. Numerous studies have demonstrated that polypyrrole has excellent photothermal properties and good biocompatibility, which endow it with great potential in oncotherapy [9–11]. In addition, photodynamic therapy (PDT) is another laser-based therapy in which photosensitizers accumulate in tumours and produce reactive oxygen species (ROS) under specific laser irradiation to kill tumour cells [12, 13]. PDT, as a rapidly developing treatment technology, has shown many advantages in the clinical treatment of various tumours, with the characteristics of reduced invasiveness, fewer side effects, and lower drug resistance [14]. Methylene blue (MB) is a heterocyclic aromatic dye soluble in ethanol or water belonging to the thiophenazine family that acts as a highly efficient photosensitizer for PDT. Another advantage of MB in biomedical fields is its good ability to penetrate across the cell membrane due to its benzene ring, resulting in its accumulation in mitochondria, lysosomes and double-stranded DNA [15, 16]. Mounting evidence has shown that the combination of PTT and PDT exhibits superior therapeutic effects in eradicating local tumours [17–19].

Recently, the effect of phototherapy on the immune system has gradually become a hot research topic owing to its immune activation behaviour. The possible mechanism of action is that phototherapy causes immunogenic cell death (ICD) in tumour cells in situ to release tumour antigens, endogenous adjuvants, heat shock proteins, and damage-associated molecular patterns (DAMPs) [20, 21]. These molecules, as ‘eat me’ signals, are recognized and expressed by antigen-presenting cells (APCs) and induce an antitumour immune response. During this process, dendritic cells (DCs), as the strongest APCs, exert the pivotal role in major histocompatibility complex (MHC)-mediated antigen cross presentation [22, 23]. Li et al. constructed a polydopamine (PDA)-based core–shell nanoplatform loaded with CpG ODNs that has a remarkable synergistic treatment effect on inducing the maturation of DCs and the activation of T cells when combined with PTT [24]. Our previous studies reported a novel NIR-responsive in situ tumour vaccine (HA-PDA@IQ/DOX HG) that promoted DC maturation, memory T-cell generation in lymph nodes and cytotoxic T lymphocyte production in the spleen after photothermal ablation [25]. However, the techniques used in these studies suffer from many bottlenecks, such as complex preparation, insufficient time and inefficient immune activation.

Herein, we report a simple two-step method for the preparation of MB@OVA/PPY NPs to achieve ideal phototherapy and an intense immune activation response. First, ovalbumin and polyvinyl alcohol (PVA) were chosen as precursors to produce OVA/PPY NPs by simple iron cation-mediated oxidative polymerization. Second, MB was loaded onto the surface of the OVA/PPY NPs via π–π stacking interactions to obtain MB@OVA/PPY NPs. The chemical and physical properties of MB@OVA/PPY NPs were characterized, and the corresponding photothermal conversion efficiency was calculated. Then, MB@OVA/PPY NPs were used as dual photosensitizers to assess their influence on the growth of tumour cells. The ability of MB@OVA/PPY NPs to induce dendritic cell (DC) maturation was studied in vitro using DC2.4 cells. In addition, a specific peptide (SIINFEKL-H-2 Kb) derived from OVA was chosen as an indicator to determine the effect of MB@OVA/PPY NPs on the antigen presentation ability of DCs. Finally, MB@OVA/PPY NPs were injected into the tumour site to investigate their influence on tumour growth and evaluate DC maturation and antigen cross presentation, cytotoxic T lymphocytes (CTLs) and memory T cells in the major peripheral immune organs.

## Materials and methods

### Materials

Polyvinyl alcohol (PVA), FeCl_3_·6H_2_O, pyrrole (Py), and methylene blue (MB) were purchased from Shanghai Aladdin Reagent Co., Ltd. (China). OVA was purchased from Shanghai Sangon Biotechnology Development Co., Ltd. (China). All reagents were used as received without further purification. Deionized water was used in the experiments.

### Synthesis of MB@OVA/PPY NPs

MB@OVA/PPY NPs were prepared based on previous publications with the appropriate modifications [7, 15]. Briefly, 0.5 g of PVA was dissolved in 10 mL of deionized water and then heated to 70 °C for 30 min to obtain a uniformly viscous solution. After cooling to room temperature, 0.5 g of OVA and 0.5 g of FeCl_3_·6H_2_O were dissolved in PVA solution and stirred for 30 min. Then, 140 μL of pyrrole was added dropwise and the mixture was stirred for 4 h. The supernatant was recovered by centrifugation at 2500 RPM for 10 min and packaged in a dialysis bag (MW = 3500 Da) to remove residual unreacted reagents. The purified solution underwent a typical lyophilization process for 48 h to obtain the OVA/PPY NP powder. Next, 0.5 mg of methylene blue was added to 20 mL of OVA/PPY NP solution (0.25 mg/mL) and stirred for approximately 24 h. Finally, MB@OVA/PPY NPs were collected after dialysis and lyophilization.

### Characterization

The particle size and morphology of the MB@OVA/PPY NPs were analysed using a Zetasizer particle size analyser and transmission electron microscopy (TEM). The elemental composition of the MB@OVA/PPY NPs was characterized by EDX spectroscopy. UV–Vis spectrophotometry was used to record the absorption spectrum. The chemical structure and surface groups of the MB@OVA/PPY NPs were detected by Fourier transform infrared (FTIR) spectroscopy. The components and contents of the MB@OVA/PPY NPs were detected by X-ray photoelectron spectroscopy (XPS). The loading of OVA into the MB@OVA/PPY NPs was determined using a MERITON SMA1000 ultramicro UV spectrophotometer.

### OVA loading and release

To investigate the amount of OVA loaded into the MB@OVA/PPY NPs, 1 mL of nanoparticles suspension in a dialysis bag (MWCO 3500 Da) was immersed in 10 mL of DI water under magnetic stirring to remove interference from MB for detection. After 24 h of dialysis, the OVA content in the MB@OVA/PPY NP solution was measured with an ultramicro UV spectrophotometer to calculate the OVA loading rate in MB.

The in vitro profiles of OVA release from the MB@OVA/PPY NPs were investigated. Briefly, 1 mL of nanoparticles suspension in a dialysis bag (MWCO 500 kDa) was immersed in 10 mL of different PBS solutions (pH 5.4, 6.4, 7.2) under magnetic stirring. At predetermined time points, 1 mL of PBS was collected, and an equal volume of fresh PBS. was added back. The content of OVA was measured with an ultramicro UV spectrophotometer.

### Photothermal performance

Different concentrations of MB@OVA/PPY NP aqueous solutions (200, 400, 600,800 mg/mL) were irradiated with 808 nm near-infrared light at different power densities (0.5, 1.0, 1.5, 2.0, 2.5 W/cm^2^). The solution temperatures were measured with a hand-held near-infrared thermal camera. Solution temperatures were recorded every 30 s. Additionally, the photothermal reproducibility and stability of the MB@OVA/PPY NPs were studied by using five laser on/off cycles. The photothermal conversion efficiency was calculated by plotting the linear regression data of a heating and cooling cycle.

### Fluorescent staining

4T1 cells in suspension (1.0 × 10^4^ cells) were seeded onto a 24-well plate. The cells were washed with PBS 3 times at 37 °C in a 5% CO_2_ incubator overnight. FITC-labelled MB@OVA/PPY NPs (100 µg/mL) were transferred to the 24-well plate and washed with PBS 3 times after incubation. Mitochondria, lysosomes and endoplasmic reticulum were stained with a red Tracker fluorescent probe. After washing with PBS 3 times, the 4T1 cells were fixed using paraformaldehyde, and a solution of DAPI dye was added. A laser scanning confocal microscope (Leisa SP8SSTED3X) was used for cell observation after washing with PBS 3 times.

### Photodynamic performance

Under 660 nm laser irradiation, the released MB could be utilized as a competent PDT photosensitizer to produce singlet oxygen (^1^O_2_) and induce cell death. Singlet oxygen (^1^O_2_) generated from the MB@OVA/PPY NPs was evaluated by detecting the absorption intensity of the ROS indicator 1,3-diphenylisobenzofuran (DPBF) at 410 nm [26]. Different concentrations of MB@OVA/PPY NP aqueous solutions (100, 200, 300, 400, 500 μg/mL) in DPBF mixed with H_2_O_2_ (as an O_2_ source) were irradiated with a 660 nm laser (50 mW/cm^2^) for 5 min. Afterwards, the absorbance of the solution at 410 nm was measured by UV–vis spectrophotometry.

### Biocompatibility

The haemocompatibility of the MB@OVA/PPY NPs was assessed with a haemolytic assay. Briefly, the MB@OVA/PPY NPs at different concentrations (50, 100, 200, 400 μg/mL) and mouse blood were coincubated for 2 h. Water was used as the positive control, and PBS was used as the negative control. The UV–Vis absorbance of the supernatant at 541 nm was measured to calculate the haemolysis rate.

The cytocompatibility of MB@OVA/PPY NPs was determined with a cell counting-8 kit (CCK-8) assay. A total of 1.0 × 10^4^ mouse embryonic fibroblast (MEF) cells and 4T1 cells (mouse breast cancer cells) were inoculated into 96-well plates for 24 h. Then, different concentrations of MB@OVA/PPY NPs (0, 50, 100, 200, 400, 800 μg/mL) were added for 48 h. After CCK-8 reagent was added, the absorbance at 450 nm in each well was measured with an enzyme plate analyser.

### Photothermal and photodynamic therapy in vitro

MEFs and 4T1 cells in suspension (1.0 × 10^4^ cells) were seeded onto a 96-well plate. After incubation for 24 h at 37 °C, medium containing different concentrations of MB@OVA/PPY NPs (0, 50, 100, 200, 400, 800 μg/mL) was added. To achieve the desired effect, the aperture of the 808 nm near-infrared laser light was adjusted to match the edge of each well of the plate. Each experimental group was irradiated with the laser at a power density of 2.0 W/cm^2^ for 5 min. Then, these cells were washed with PBS and placed in an incubator overnight. Next, 10 μL of CCK-8 reagent was added to each well for incubation for 2 h. The absorbance values of each well at 450 nm were measured with an enzyme-labelled analyser to calculate cell viability. For PDT assessment, the procedure was similar to that for PTT assessment. 4T1 cells were cultured with different concentrations of MB@OVA/PPY NPs (0, 50, 100, 200, 400 μg/mL). Photodynamic therapy was conducted by using a 660 nm laser (50 mW/cm^2^) for 5 min. A CCK-8 assay was carried out to determine cell viability.

### Maturation and antigen presentation capability of DCs in vitro

DC2.4 cells were chosen to characterize the immune induction functions of MB@OVA/PPY NPs in vitro. First, DC2.4 cells were incubated with different concentrations of MB@OVA/PPY NPs (10–400 μg/mL) for 24 h, stained with an apoptosis kit (Annexin V-FITC/PI) for 30 min, and then washed, and the cell activity was detected by flow cytometry. Then, the DC2.4 cells were coincubated with MB@OVA/PPY NPs (10–400 μg/mL) for 24 h. These cells were washed and stained with anti-CD86-PE and anti-CD80-FITC for 30 min. The cells were washed with FACS buffer and sorted by flow cytometry [27].

The antigen cross-presentation ability of DCs was determined by detecting the expression profile of SIINFEKL-H-2 kb on the cell surface. DC2.4 cells were incubated with different concentrations of MB@OVA/PPY NPs (10, 50, 100 μg/mL) for 24 h. Three replicates were set in each group, and flow cytometry was used for detection.

### Mouse tumour model

Female BALB/c mice were purchased from Jiangsu ALF Biotechnology Co., Ltd. Animal experiments were carried out in accordance with the protocol approved by the Experimental Animal Center of Jiangsu University. To establish a tumour model, 4T1 cells (1 × 10^6^) in 50 μL of PBS were subcutaneously injected into the lower left breast of each mouse. For in vivo combination therapy, 4T1 tumour-bearing mice were divided into four groups, including the PBS, PPY NP, MB@PPY NP, and MB@OVA/PPY NP groups. After approximately one week, the average sizes of the tumours reached approximately 100 mm^3^, and then each mouse in each group received approximately 1 mg of nanoparticles via in situ injection. The tumours were irradiated with an 808 nm laser at a power density of 2 W/cm^2^ for 5 min and then irradiated with a 660 nm laser at a power density of 100 mW/cm^2^ for 5 min. During laser irradiation, the temperature changes in the tumours were recorded with an infrared thermal imaging camera (HT-19). After treatment, tumour size was monitored with a vernier calliper, and the lengths and widths were recorded every two days for two weeks. The tumour volumes were calculated by the formula length × width^2^/ 2.

### Measurement of the memory T cell, DC and CD8+ T-cell populations

After challenging mice with 4T1 tumours for seven days, the inguinal lymph nodes and spleens were isolated and dissociated into single cells by mashing through cell strainers (70 μm). The inguinal lymph node and spleen cell suspensions were stained with APC anti-mouse CD3 (Biolegend), PE anti-mouse CD8a (Biolegend), PE/Cy7 anti-mouse CD62L (Biolegend), FITC anti-mouse/human CD44 (Biolegend), APC anti-mouse CD11c, FITC anti-mouse CD80, PE anti-mouse CD86 and APC anti-mouse CD3, and PE anti-mouse CD8a. The percentages of CD3+CD8+CD44+CD62− cells, CD11c+CD80+CD86+ cells, and CD3+CD8+ cells corresponding to effector memory T cells, DCs, and CD8+ T cells, were analysed by flow cytometry.

## Results and discussion

### Preparation and characterization of the MB@OVA/PPY NPs

**Scheme 1:**
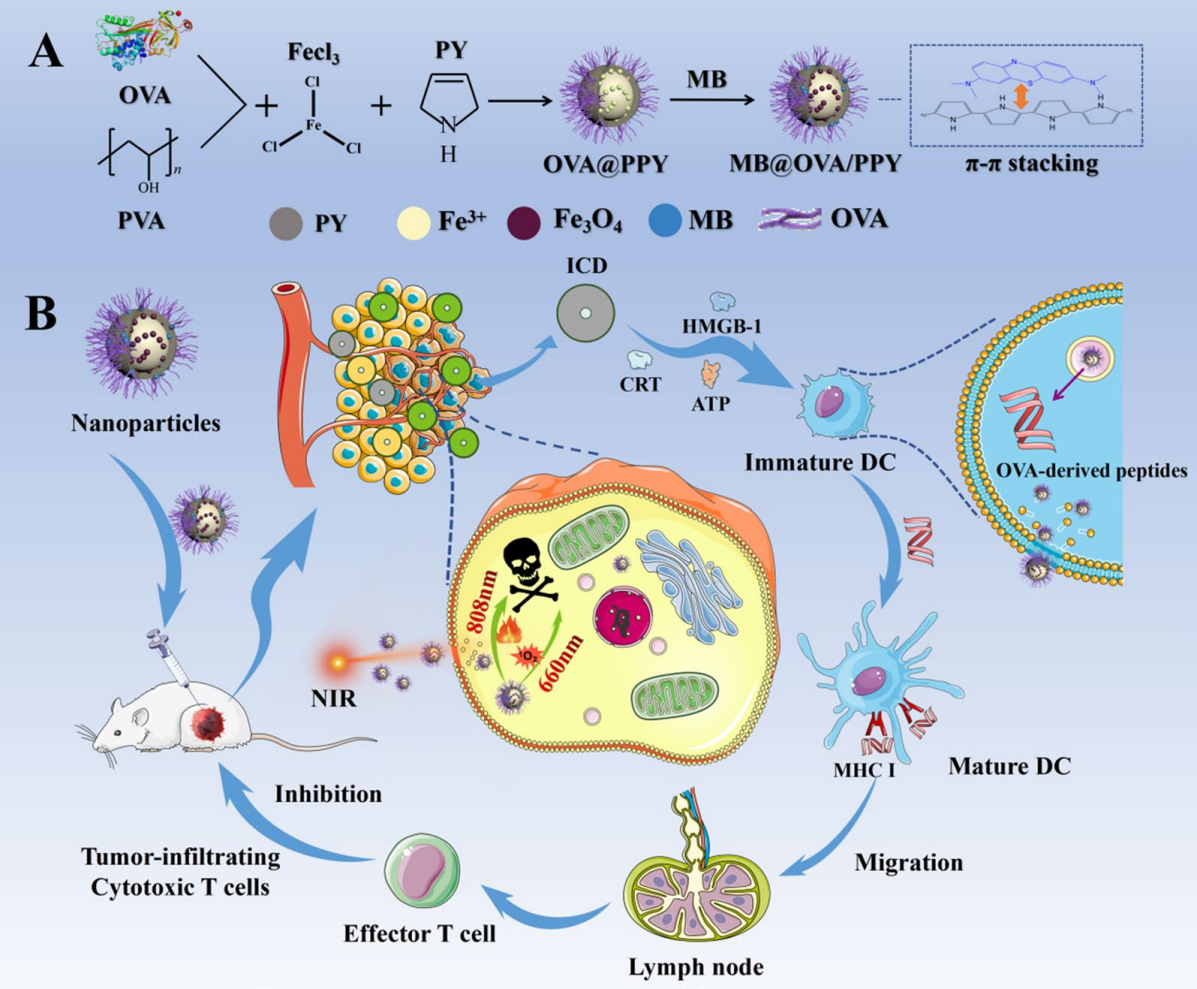
Schematic diagram of the preparation (**A**) and subsequent application (**B**) of MB@OVA/PPY NPs

**Fig. 1:**
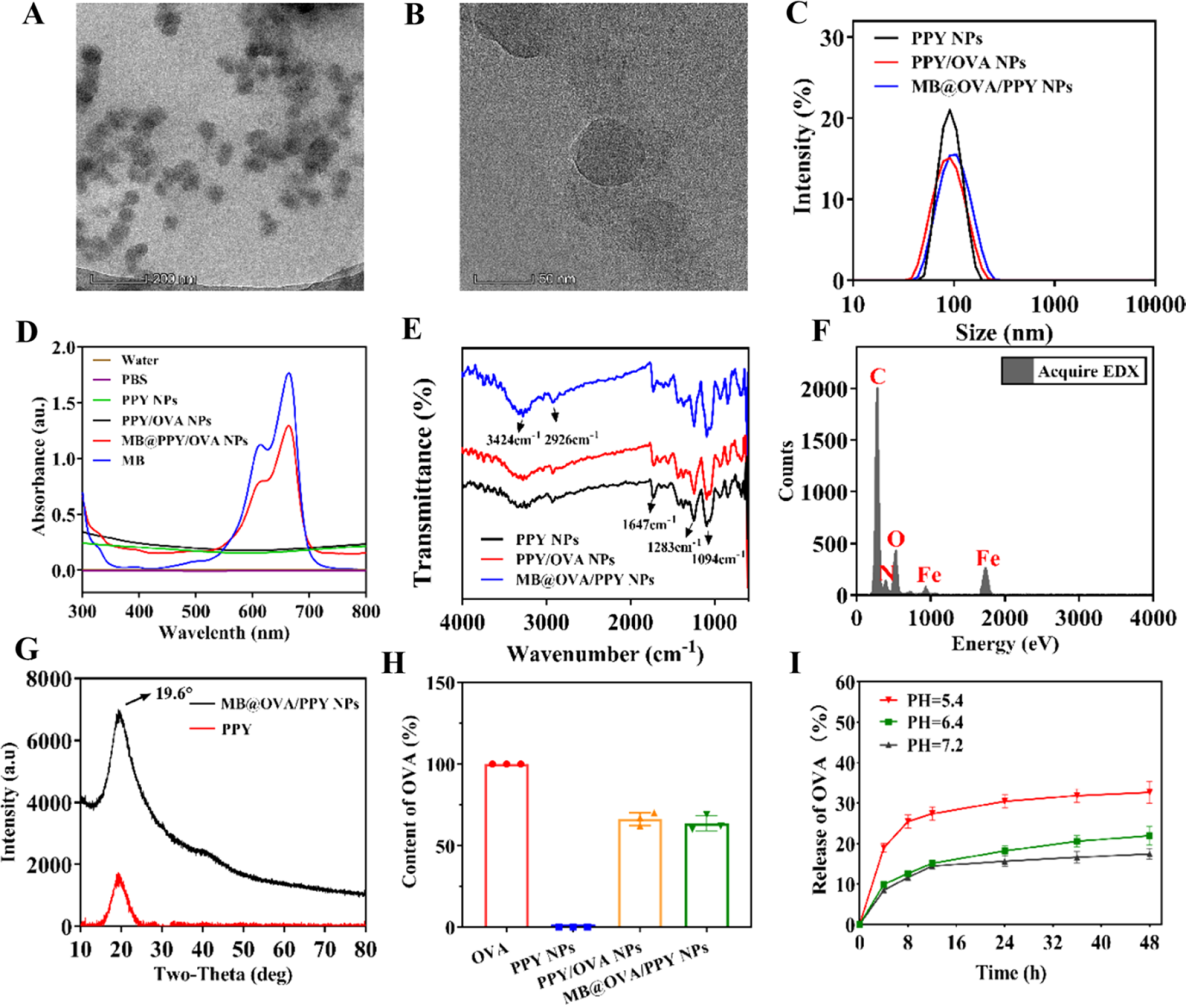
Characterization of the MB@OVA/PPY NPs. **A**, **B** TEM image. **C** Particle size distribution. **D** UV–Vis absorbance spectra. **E** FTIR spectra. **F** EDX spectrum. **G** XRD spectrum. **H** Content of OVA. **I** Release of OVA at different pH values

MB@OVA/PPY NPs were successfully prepared using the two-step method in Scheme 1. First, the biological macromolecule OVA was integrated into the polypyrrole nanoparticles by facile iron cation-mediated oxidative polymerization using water-soluble polyvinyl alcohol (PVA) as a stabilizer and FeCl_3_ as an oxidizing agent. Then, the photosensitizer MB was loaded via π–π stacking interactions to form MB@OVA/PPY NPs. In Fig. 1A and B, the TEM images show that the MB@OVA/PPY NPs were spherical and uniformly dispersed without obvious aggregation. Little effect on particle size was observed after loading OVA and MB. As shown in Fig. 1C, the MB@OVA/PPY NPs had an average particle size of 98 nm, which was similar to that of the PPY NPs and PPY/OVA NPs. The loading of methylene blue on the surface of the polypyrrole nanoparticles was verified using the UV–Vis spectroscopy. Figure 1D shows that the specific absorption peak of methylene blue appeared at 660 nm, and this peak was absent in the spectra of the unloaded PPY NPs and PPY/OVA NPs. As we expected, there were clear, specific absorption peaks from MB in the MB@OVA/PPY NP spectrum at 660 nm. These data indicated that methylene blue was successfully loaded on the surface of the PPY/OVA NPs.

The chemical composition of the MB@OVA/PPY NPs was investigated using FTIR and EDX spectroscopy. In Fig. 1E, the FTIR spectra of MB@OVA/PPY NPs showed that a wide absorption peak appeared near 3400 cm^−1^, which was ascribed to the –OH stretching vibration. The asymmetric stretching vibration peak from –CH_2_ appeared near 2926 cm^−1^, and the peak at 1647 cm^−1^ belonged to the scissoring vibration of C=C. The peak near 1283 cm^−1^ corresponded to the bending vibration of –CH, and the peak at 1094 cm^−1^ was ascribed to the contraction vibration of –C–O. The FTIR results showed that the surfaces of the MB@OVA/PPY NPs were rich in a large number of active groups, indicating the successful fabrication of MB@OVA/PPY NPs. The hydroxyl and acyl groups endowed the MB@OVA/PPY NPs with good hydrophilicity and water dispersion properties, and the peak at 1647 cm^−1^ due to C=C stretching vibrations shifted slightly and decreased in intensity, indicating the presence of π–π interactions between MB and the PPY/OVA NPs. The EDX spectrum showed that the MB@OVA/PPY NPs were mainly composed of C, O, N and Fe, and the doping amount of Fe was approximately 5.44% (Fig. 1F). The XRD results (Fig. 1G) showed that the MB@OVA/PPY NPs had a maximum absorption peak at approximately 19.6°, which was consistent with polypyrrole.

The OVA loading rate in the MB@OVA/PPY NPs was also studied. Figure 1H shows that the OVA loading rate in the PPY/OVA NPs was approximately 70%, indicating that OVA was successfully loaded into the nanoparticles and that the loading efficiency was satisfactory. There was no significant OVA release during MB loading, which shows that the loading of MB through π–π stacking interactions does not affect the loaded OVA. Then, the OVA release profiles were studied in vitro at pH 5.4, pH 6.4 and pH 7.2 to investigate the pH-responsiveness of the MB@OVA/PPY NPs. From Fig. 1I, we can see that the MB@OVA/PPY NPs exhibited pH-responsive OVA release behaviour. At pH 7.2, the MB@OVA/PPY NPs showed low OVA release, with approximately 12% cumulative release within 48 h. In contrast, OVA release was significantly accelerated at lower pH, and the cumulative release was greater than 30% at pH 5.4, which might be attributed to the cleavage of the coordination bond between Fe and OVA.

**Fig. 2:**
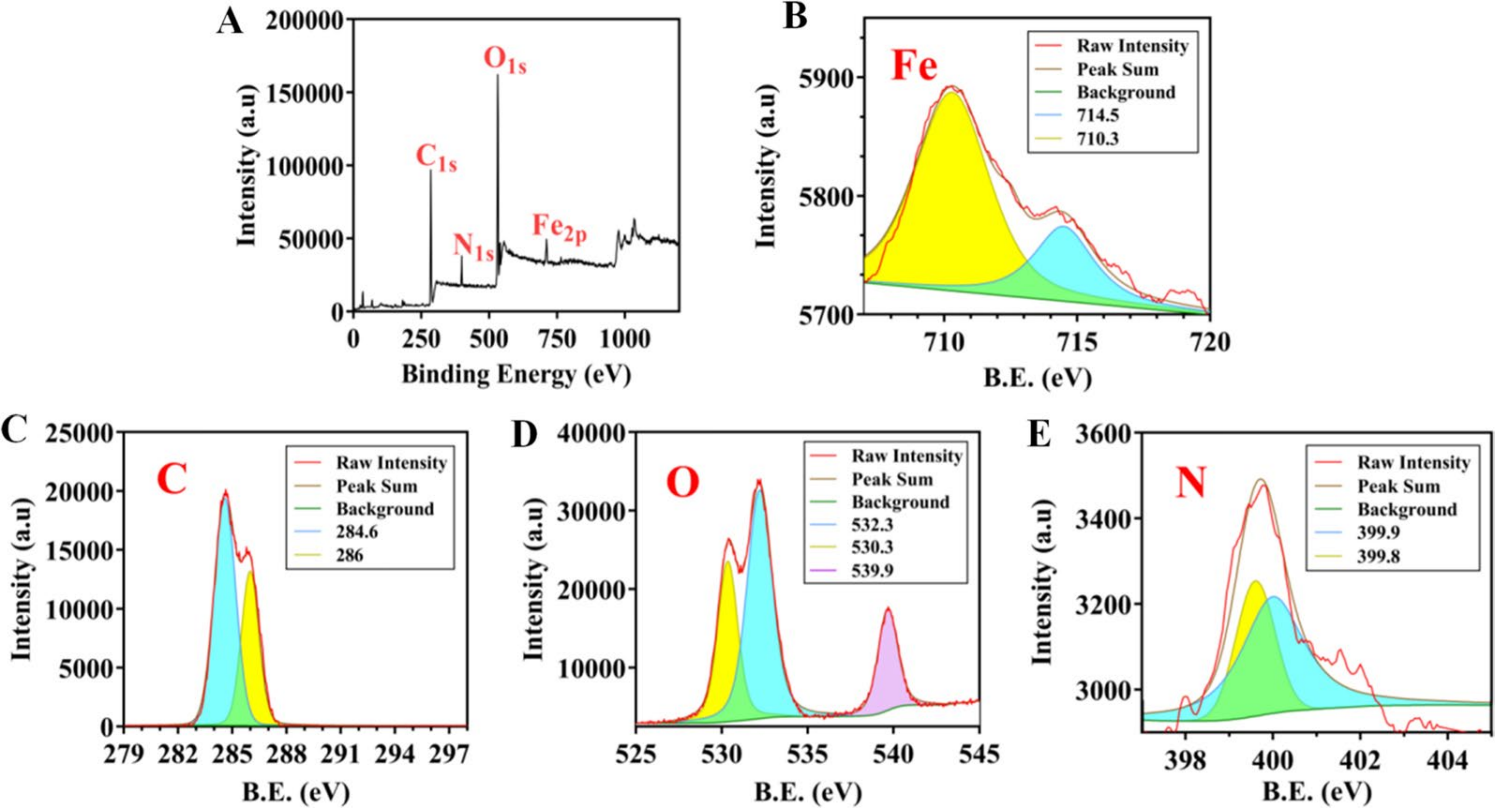
**A** Full-scan XPS spectrum of the MB@OVA/PPY NPs. **B** Fe_2p_ spectrum. **C** C_1s_ spectrum. **D** O_1s_ spectrum. **E** N_1s_ spectrum

The XPS results (Fig. 2A) showed four peaks that appeared near 710 eV, 530 eV, 399 eV and 284 eV, corresponding to the four elements Fe, C, O and N, respectively. The Fe_2p_ XPS spectrum suggested that the MB@OVA/PPY NPs contained mixed valence state iron (Fe^3+^ and Fe^2+^). As shown in Fig. 2B, the peak at 710 eV indicated the presence of Fe^3+^, and the peak at 714.5 eV indicated the presence of Fe^2+^. Additionally, the peak at 710 eV was stronger than that at 714.5 eV, which proved that the bound Fe in the MB@OVA/PPY NPs mainly existed in the trivalent form. The C_1s_ spectrum (Fig. 2C) showed two main peaks at 286 eV and 284.6 eV, which corresponded to C–C/C–O and C=C, respectively. The O_1s_ spectrum (Fig. 2D) had three distinct peaks at 532.3 eV, 530.3 eV and 539.9 eV, which were caused by O-C, Fe_3_O_4_ and H_2_O. The N_1s_ spectrum (Fig. 2E) showed two peaks at 399.9 eV and 399.8 eV, indicating the presence of nitrogen atoms [28, 29]. The above data were consistent with our expectations, which proved that the prepared MB@OVA/PPY NPs contained Fe, C, O and N.

### Photothermal conversion performance and photodynamic behaviour

**Fig. 3:**
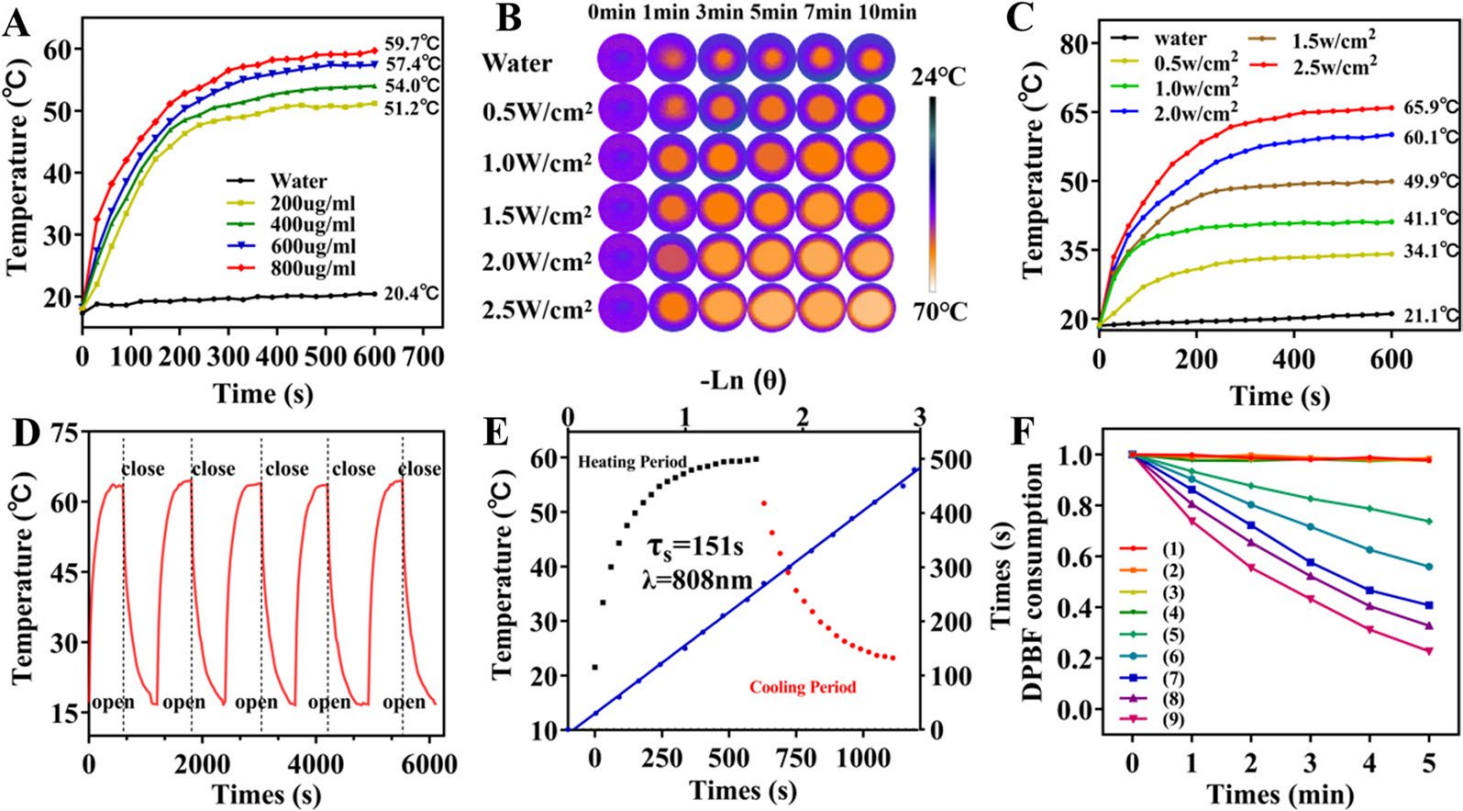
**A** Photothermal heating curves of various concentrations of MB@OVA/PPY NP solution under 808 nm NIR laser irradiation at a power density of 2 W/cm^2^. **C** Photothermal heating curves and **B** the corresponding infrared thermal image of the MB@OVA/PPY NP solution (800 μg/mL) under 808 nm NIR laser irradiation with varied power densities. **D** Heating curves of the MB@OVA/PPY NP solution for five laser on/off cycles under irradiation with an 808 nm NIR laser (2 W/cm^2^). **E** Photothermal effect of the MB@OVA/PPY NP solution under 808 nm NIR laser irradiation (black dots) Red dots indicate when the laser was turned off. The time constant (τ_s_) for the heat transfer from the system was determined by applying the linear time data from the cooling period (blue line). (F) ^1^O_2_ production efficiency of the different samples with different treatments (1. DPBF, 2. DPBF + laser, 3. DPBF + H_2_O_2_, 4. DPBF + H_2_O_2_ + laser, 5–9. DPBF + laser + MB@OVA/PPY NPs at 100, 200, 300, 400, 500 μg/mL, respectively)

The photothermal conversion performance of the MB@OVA/PPY NPs was characterized under 808 nm NIR irradiation. As shown in Fig. 3A, the temperature of the MB@OVA/PPY NP solution increased gradually with increasing NP concentration (200–800 μg/mL) under 808 nm NIR laser irradiation at a power density of 2.0 W/cm^2^. The maximum temperature in each group increased from 51.2 °C (200 μg/mL) to 59.7 °C (800 μg/mL) after 10 min of irradiation. Subsequently, the MB@OVA/PPY NP solution (800 μg/mL) was irradiated with NIR irradiation at different power densities (0.5, 1.0, 1.5, 2.0, 2.5 W/cm^2^). Figure 3C shows that under these conditions, there was a similar gradual temperature increase. When the power density reached 2.5 W/cm^2^, the peak temperature exceeded 65.9 °C. The photothermal reproducibility and stability of the MB@OVA/PPY NPs were tested by switching the laser on and off. The results showed that the temperature of the MB@OVA/PPY NP aqueous solution rose rapidly to higher than 60 °C after the laser was switched on and dropped rapidly after the laser was switched off. The obtained temperature curves matched each other well over five on/off cycles (Fig. 3D). The above results indicated that the MB@OVA/PPY NPs had reliable photothermal conversion stability and reproducibility. Finally, through execution of a heating–cooling cycle, linear regression was used to calculate that the integrated photothermal conversion efficiency of the MB@OVA/PPY NPs was 38% at 808 nm, which was higher than that of the previously reported PTT agents [7].

Methylene blue (MB) is a heterocyclic aromatic dye belonging in the thiophenazine family that can be utilized as an efficient PDT photosensitizer for generating singlet oxygen (^1^O_2_), which is responsible for PDT-mediated cell death. Therefore, the ROS indicator DPBF was used to evaluate the capability of MB@OVA/PPY NPs to generate ^1^O_2_ under 660 nm laser irradiation. The light absorbed by DPBF decreased in a stepwise manner with increasing concentrations of MB@OVA/PPY NPs. The maximum light absorption value of DPBF decreased by approximately 73% after 5 min of 660 nm laser irradiation at 50 mW/cm^2^ (Fig. 3F) at a NP concentration of 500 μg/mL These data indicated that the MB@OVA/PPY NPs had a good ability to produce ROS, which was proportional to the NP concentration and time of exposure. Based on these results, we speculated that MB@OVA/PPY NPs might be used as ideal photosensitizers for photodynamic therapy of tumours.

**Fig. 4:**
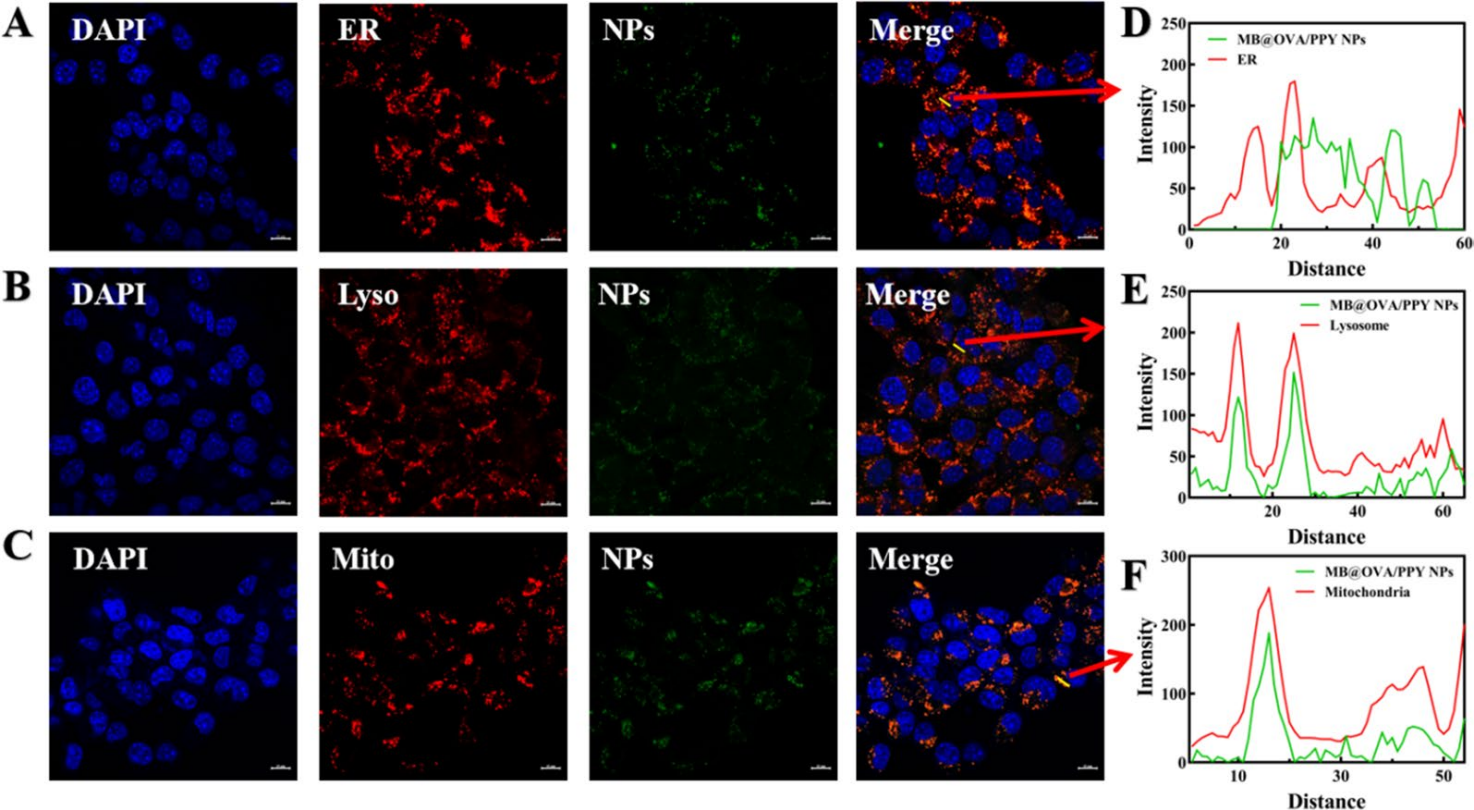
LSCM images of 4T1 cells treated with MB@OVA/PPY NPs-FITC for 6 h. The nucleus and organelles (endoplasmic reticulum, lysosomes, mitochondria) were stained with DAPI and specific organelle probes (ER, Lyso, Mito), respectively. The intensity profile within the regions of interest (yellow line in each Merge image) of the MB@OVA/PPY NPs (green line) and the specific organelle probe (red line) is shown on the right. Scale bar: 10 µm

### Intracellular distribution of MB@OVA/PPY NPs

The uptake and colocalization of MB@OVA/PPY NPs in cells were characterized using confocal fluorescence microscopy. As shown in Fig. 4, bright green fluorescence was observed in the cytoplasm of each group after 6 h of incubation, indicating that FITC-labelled MB@OVA/PPY NPs could be easily internalized by cells. To further explore their distribution in the cytoplasm, three kinds of fluorescent (ER, Lyso, Mito) probes were chosen to label specific organelles. Through greyscale analysis of the images, we found that the MB@OVA/PPY NPs overlapped with the grey levels of lysosomes and mitochondria but had poor overlap with the endoplasmic reticulum, indicating that the MB@OVA/PPY NPs were more easily localized to the lysosomes and mitochondria. A possible reason for this may be that the internalized MB@OVA/PPY NPs readily entered the lysosomes and then spread into the mitochondria through the lysosomal escape pathway. These findings revealed that MB@OVA/PPY NPs were mainly distributed in the lysosomes and mitochondria after endocytosis.

**Fig. 5:**
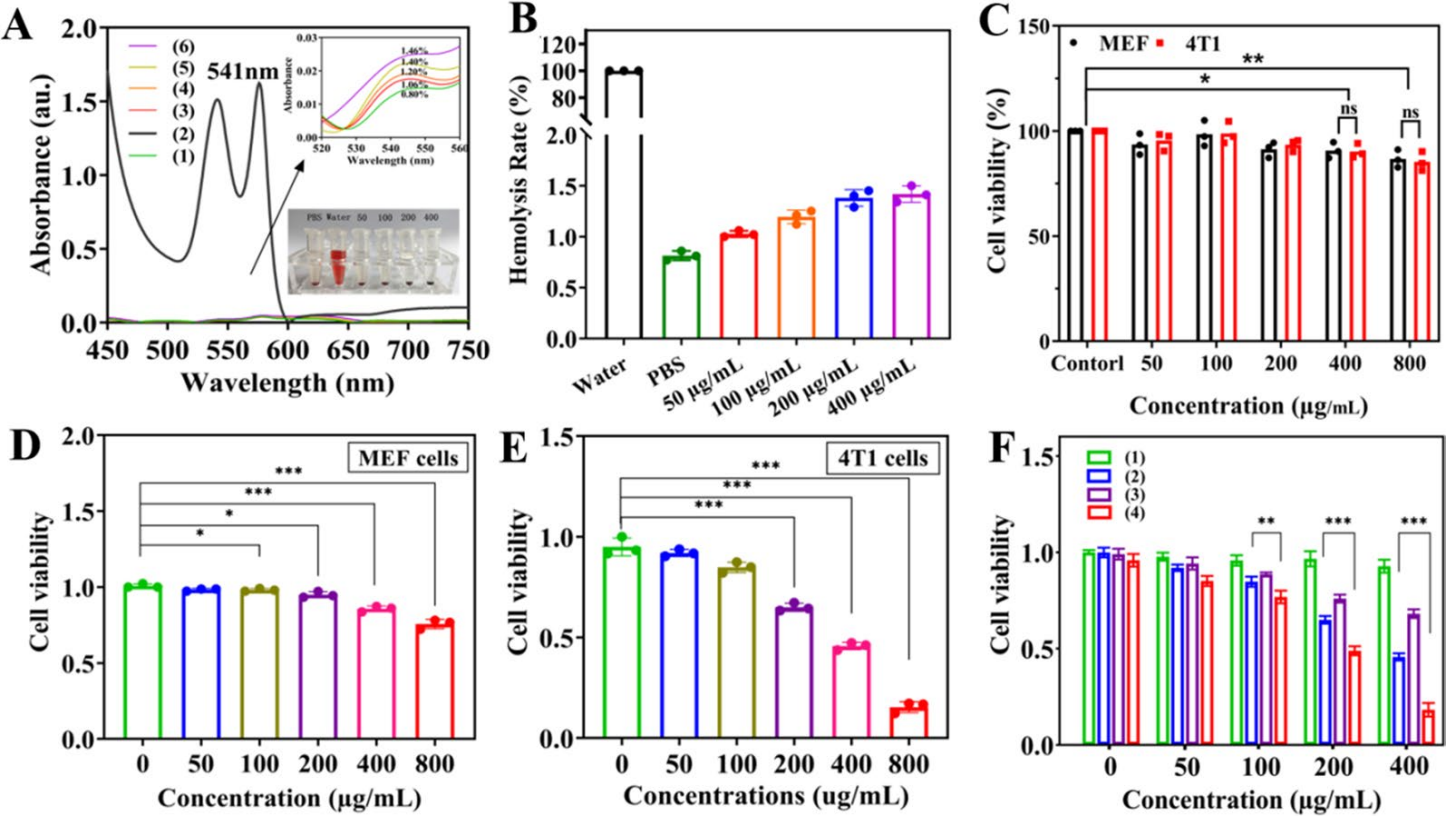
**A**, **B** Haemolytic profile of red blood cells after incubation with various concentrations of MB@OVA/PPY NPs for 2 h (1, PBS; 2, water; 3–5, 50–400 µg/mL NPs). **C** Cytocompatibility of the MB@OVA/PPY NPs. The effects of PTT mediated by MB@OVA/PPY NPs on MEF (**D**) and 4T1 cell (**E**) viability under 808 nm NIR irradiation at a power intensity of 1.0 W/cm^2^ for 5 min. **F** The effect of combined photothermal and photodynamic treatment mediated by MB@OVA/PPY NPs on 4T1 cell viability in vitro (1. Control; 2. 808 nm laser; 3. 660 nm laser; 4. 808 nm + 660 nm laser). p values were calculated by Student’s t test. Values are reported as the means ± SD (n = 3) of three independent experiments, *p < 0.05, **p < 0.01, ***p < 0.001, ****p < 0.001

### Biocompatibility of the MB@OVA/PPY NPs

The biocompatibility of biomedical materials is essential for clinical applications. Therefore, a haemolytic test was conducted to assess the blood compatibility of the MB@OVA/PPY NPs. Briefly, different concentrations of MB@OVA/PPY NP solution were coincubated in mouse blood for 2 h. Compared to PBS treatment, no significant haemolysis of red blood cells was observed after treatment with increasing concentrations (0–400 µg/mL) of MB@OVA/PPY NPs (Fig. 5A, B). When the NP concentration was 400 µg/mL, the haemolysis rate was only 1.46%. This result indicated that the MB@OVA/PPY NPs had good blood compatibility.

Next, the cytotoxicity of the MB@OVA/PPY NPs was evaluated using standard CCK-8 assays in normal cells (MEFs) and cancer cells (4T1 cells). Figure 5C shows that the viability of both types of cells was not significantly affected after incubation with the MB@OVA/PPY NPs for 48 h over a wide concentration range (0, 50, 100, 200, 400, 800 µg/mL). Even at the high NP concentration of 800 µg/mL, the viability of both types of cells remained at approximately 80%. These findings indicated that the MB@OVA/PPY NPs possessed favourable cytocompatibility and could be used as alternative biomaterials for biomedical applications in vitro and in vivo.

### PTT/PDT performance of the MB@OVA/PPY NPs

The PTT therapeutic effect of the MB@OVA/PPY NPs was explored by measuring cell viability under 808 nm NIR irradiation. As the concentration of MB@OVA/PPY NPs increased, the viabilities of both types of cells gradually decreased under 1.0 W/cm^2^ irradiation. Although there was a similar concentration-dependent effect, 4T1 cell viability showed a more obvious decreasing trend than MEF viability (Fig. 5D, E). When the NP concentration reached 800 µg/mL, MEFs still displayed a 74% survival rate, while 4T1 cells had only a 15% survival rate. These findings indicated that the MB@OVA/PPY NPs had good photothermal performance to inhibit tumour cell growth. In addition, the sensitivity of the tumour cells to temperature might be attributed to the high metabolic activity of tumour cells. This phenomenon was beneficial to reduce the damage to normal tissues caused by PTT and enhance the selective killing of tumour cells.

Afterwards, the combined photothermal and photodynamic performance of the MB@OVA/PPY NPs was investigated to evaluate their tumour killing abilities. 4T1 cells incubated with MB@OVA/PPY NPs were irradiated successively with 808 nm NIR (1.0 W/cm^2^) and 660 nm (50 mW/cm^2^) lasers. When the concentration of MB@OVA/PPY NPs reached 100 µg/mL, cell viability was significantly reduced after 660 nm and 808 nm dual laser irradiation, as shown in Fig. 5F. It is worth noting that cell viability after 808 nm laser irradiation alone was lower than that after 660 nm laser irradiation alone, indicating that PTT had a better therapeutic effect than PDT. Furthermore, dual laser irradiation exhibited stronger inhibition of tumour cell viability than 660 nm or 808 nm laser irradiation alone. When the concentration of MB@OVA/PPY NPs reached 400 µg/mL, the inhibition caused by dual laser irradiation was approximately 82%, and those of 660 nm and 808 nm laser irradiation individually were 32% and 55%, respectively. Therefore, the combination of photothermal therapy and photodynamic therapy has a stronger killing effect on tumour cells, especially at low NP concentrations.

### The effect of MB@OVA/PPY NPs on DC maturation and antigen presentation in vitro

DCs are the most important APCs in the body and can not only activate the immune response but also induce immune tolerance and play an important role in maintaining the immune balance of the body. Immature DCs can take up antigens through endocytosis, and mature DCs have strong antigen presentation functions. During endocytosis, antigens are recognized and decomposed into polypeptides, and the major histocompatibility complex (MHC) signals juvenile T cells in the draining lymph nodes to induce an immune response [30]. Mature DCs express high levels of MHC class II and costimulatory molecules, such as CD40, CD80, and CD86, which provide the signals required for T-cell activation. The maturity of DCs is an important indicator reflecting the immune response during immunotherapy, which is determined by the expression levels of CD80 and CD86 on the cell membrane [27].

**Fig. 6:**
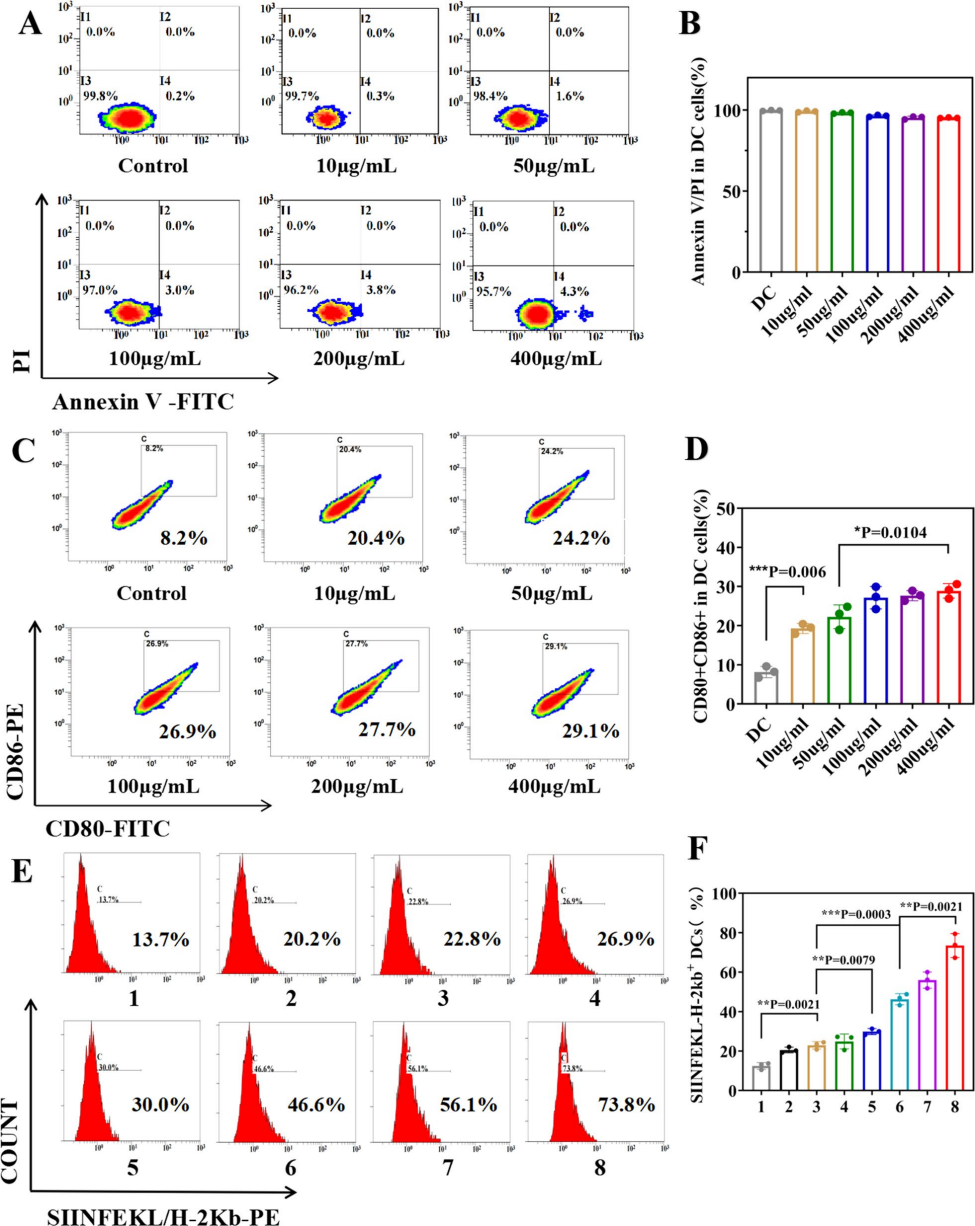
**A** Flow cytometry to evaluate the viability of DCs incubated with different concentrations of MB@OVA/PPY NPs (10, 50, 100, 200, 400 μg/mL) for 24 h and **B** the corresponding statistical data. **C** Flow cytometry analysis of the expression levels of CD80 and CD86 on the surface of DCs incubated with different concentrations of MB@OVA/PPY NPs (10, 50, 100, 200, 400 μg/mL) for 24 h and **D** the corresponding statistical data. **E** Flow cytometry analysis of SIINFEKL-H-2 Kb expression on the surface of DC2.4 cells after coculture with different treatments [1. Control; 2. OVA; 3. MB@PPY (10 μg/mL) + OVA; 4. MB@PPY (50 μg/mL) + OVA; 5. MB@PPY (100 μg/mL) + OVA; 6. MB@OVA/PPY (10 μg/mL); 7. MB@OVA/PPY (50 μg/mL); 8. MB@OVA/PPY (100 μg/mL)] and **F** the corresponding statistical data. p values were calculated by Student’s t test. Values are reported as the means ± SD (n = 3) of three independent experiments

First, considering that cells, and especially dead cells, are autofluorescent, DC viability after incubation with MB@OVA/PPY NPs should be detected before the test to ensure the reliability of the results. As shown in Fig. 6A, after incubation for 24 h with different concentrations of MB@OVA/PPY NPs, DC viability was no less than 95%. We believe that non-specific staining caused by dead cells has little effect on the results.

Therefore, we detected the effect of MB@OVA/PPY NPs on the maturation of DCs using flow cytometry. After 24 h of coincubation, the CD80 and CD86 expression levels in the MB@OVA/PPY NP group were significantly increased even at the low NP concentration of 10 µg/mL (20.4%) compared with the control group (8.2%), as shown in Fig. 6C. Moreover, CD80 and CD86 expression also increased with increasing MB@OVA/PPY NP concentration, indicating a concentration-dependent effect. These data indicated that MB@OVA/PPY NPs had the ability to promote the maturation of DCs, which is beneficial to further elicit a robust antitumour immune response in vivo.

Previous publications have reported that mature DCs have a superior antigen cross presentation ability [31, 32]. Therefore, we chose a specific peptide (SIINFEKL-H-2 Kb) derived from OVA as an indicator to detect the effect of MB@OVA/PPY NPs on the antigen cross-presentation ability of DCs. It was proven that DCs captured OVA and increased the expression level of SIINFEKL-H-2 Kb on their surface. As shown in Fig. 6E, the expression of SIINFEKL-H-2 Kb was positively correlated with the concentration of MB@OVA/PPY NPs. At the same concentration, the results in the MB@OVA/PPY NP group (46.6–73.8%) were more than twice as high as those in the free OVA group (22.8–30.0%). This result indicated that the MB@OVA/PPY NPs could notably enhance the antigen presentation ability of DCs, which might be ascribed to their larger specific surface area, increased solubility, and nanoscale size [34, 35].

### Immune response assessment in vivo

**Fig. 7:**
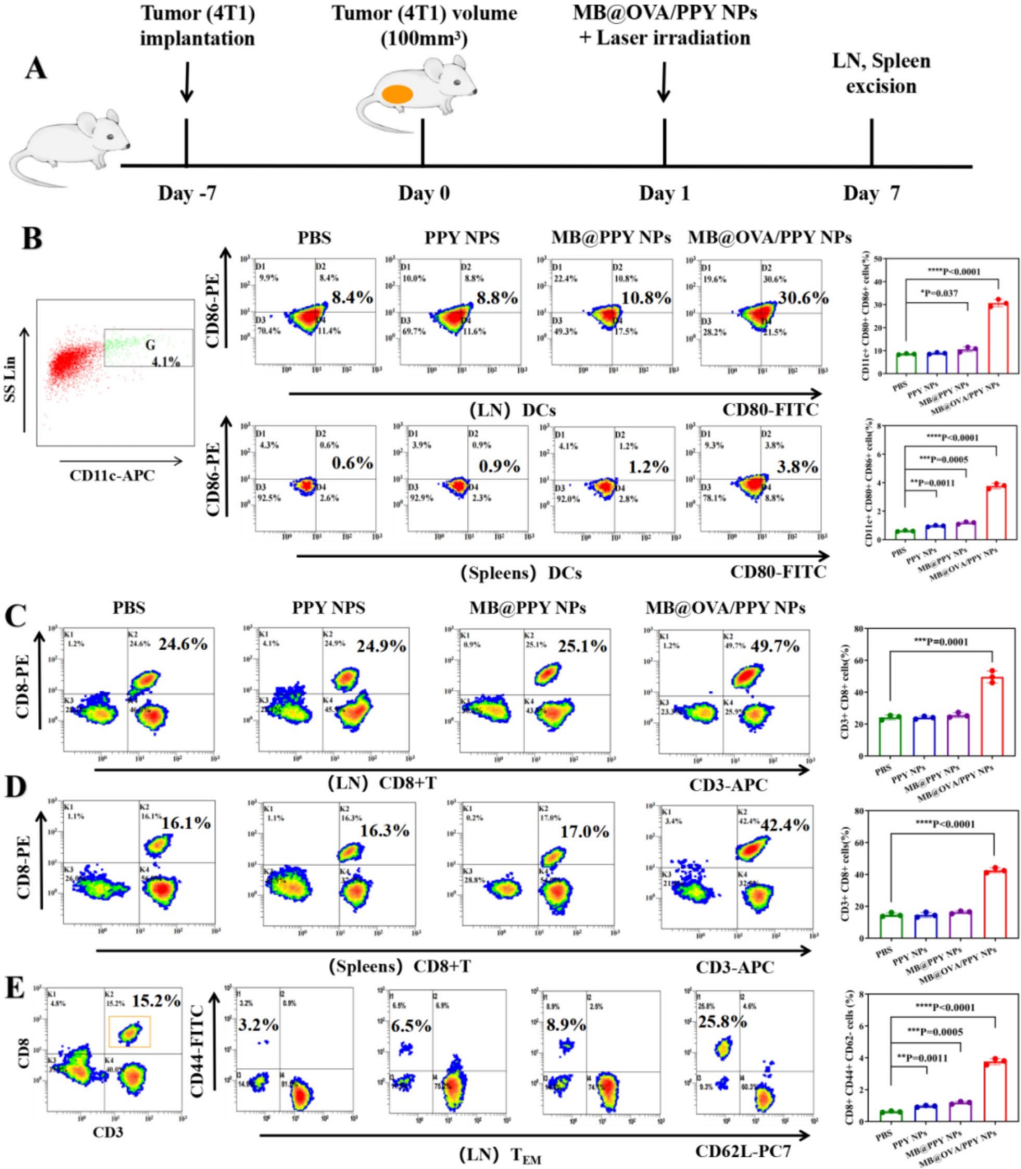
Immune response assessment in vivo. **A** Scheme and timeline of the experimental design to evaluate the in vivo immune responses triggered by MB@OVA/PY NP treatment. **B** Representative flow cytometry plots and statistical data of the CD80+ and CD86+ cells among the CD11c + DCs extracted from the inguinal lymph nodes (LNs) and spleens. **C**, **D** Representative flow cytometry plots and statistical data of the CD3+CD8+ T cells in the inguinal LNs and spleens. p values were calculated by t test. **E** Representative flow cytometry plots and statistical data illustrating the memory T cells in the inguinal LNs. p values were calculated by Student’s t test. Values are reported as the means ± SD (n = 3) of three independent experiments, *p < 0.05, **p < 0.01, ***p < 0.001, ****p < 0.001

In view of the excellent performance in vitro, we further studied the immunomodulatory functions of MB@OVA/PPY NPs in vivo. The maturation of DCs in the inguinal lymph nodes and spleens of mice with breast cancer was detected by flow cytometry. As shown in Fig. 7B, the ratios of CD11c^+^ and CD80^+^/CD86^+^ cells in the spleen after PBS, PPY NP and MB@PPY NP treatment were 0.6%, 0.9%, and 1.2%, respectively. Notably, the ratio of CD11c^+^ and CD80^+^/CD86^+^ cells in the MB@OVA/PPY NP group was 3.8%, which was higher than that in the other groups. In the lymph nodes, the ratio of CD11c^+^ and CD80^+^/CD86^+^ cells in the MB@OVA/PPY NP group (30.6%) was higher than those in the PBS (8.4%), PPY NP (8.8%) and MB@PPY NP (10.8%) groups. These in vivo data were consistent with the in vitro results, which further confirmed that the MB@PPY NPs possessed the potential to promote DC maturation in vivo.

Cytotoxic T lymphocytes (called CTLs or CD8^+^ T cells) play an essential role in the antitumour immune response, as they are activated by tumour-derived antigens and destroy target cells directly. Therefore, we analysed the CD8^+^ T cells in the lymph nodes and spleen after MB@OVA/PPY NP treatment. In this experiment, each group of inguinal lymph node cells and spleen cells was costained with anti-CD3 and anti-CD8a antibodies and measured by flow cytometry. As shown in Fig. 7B, the percentage of CD3^+^CD8^+^ T cells from the spleen after MB@OVA/PPY NP treatment (42.4%) was higher than those after treatment with PBS, PPY NPs, and MB@PPY NPs (16.1%, 16.3%, 17.0%, respectively). Moreover, the percentages of CD3^+^/CD8^+^ T cells from the inguinal lymph nodes in the PBS, PPY NP, MB@PPY NP and MB@OVA/PPY NP groups were 24.6%, 24.9%, 25.1%, and 49.7%, respectively, which showed a similar trend to that observed in the spleen. It is worth noting that the percentages of CD3^+^CD8^+^ T cells from the inguinal lymph nodes and spleen after MB@OVA/PPY NP treatment were more than two times greater than those in the other treatment groups. This enhancement in CTLs might be ascribed to the introduction of OVA, which remarkably stimulated DC maturation and enhanced the tumour-derived antigen presentation function.

Finally, we explored the effect of MB@OVA/PPY NPs on effector memory T cells (CD3^+^CD8^+^CD44^+^CD62^−^) in inguinal lymph nodes to study the potential utility of this treatment strategy for the long-term prevention of tumour recurrence. As shown in Fig. 7E, we tested memory T cells on the 7th day, the ratio of memory T cells in the PPY NP group and MB@PPY NP group improved slightly. However, the ratio of memory T cells reached 25.8% after MB@OVA/PPY NP treatment, which was more than 8 times that of the control group (PBS). These results demonstrated that MB@OVA/PPY NPs have the ability to promote memory T-cell production.

### Antitumour effect of MB@OVA/PY NPs in vivo

**Fig. 8:**
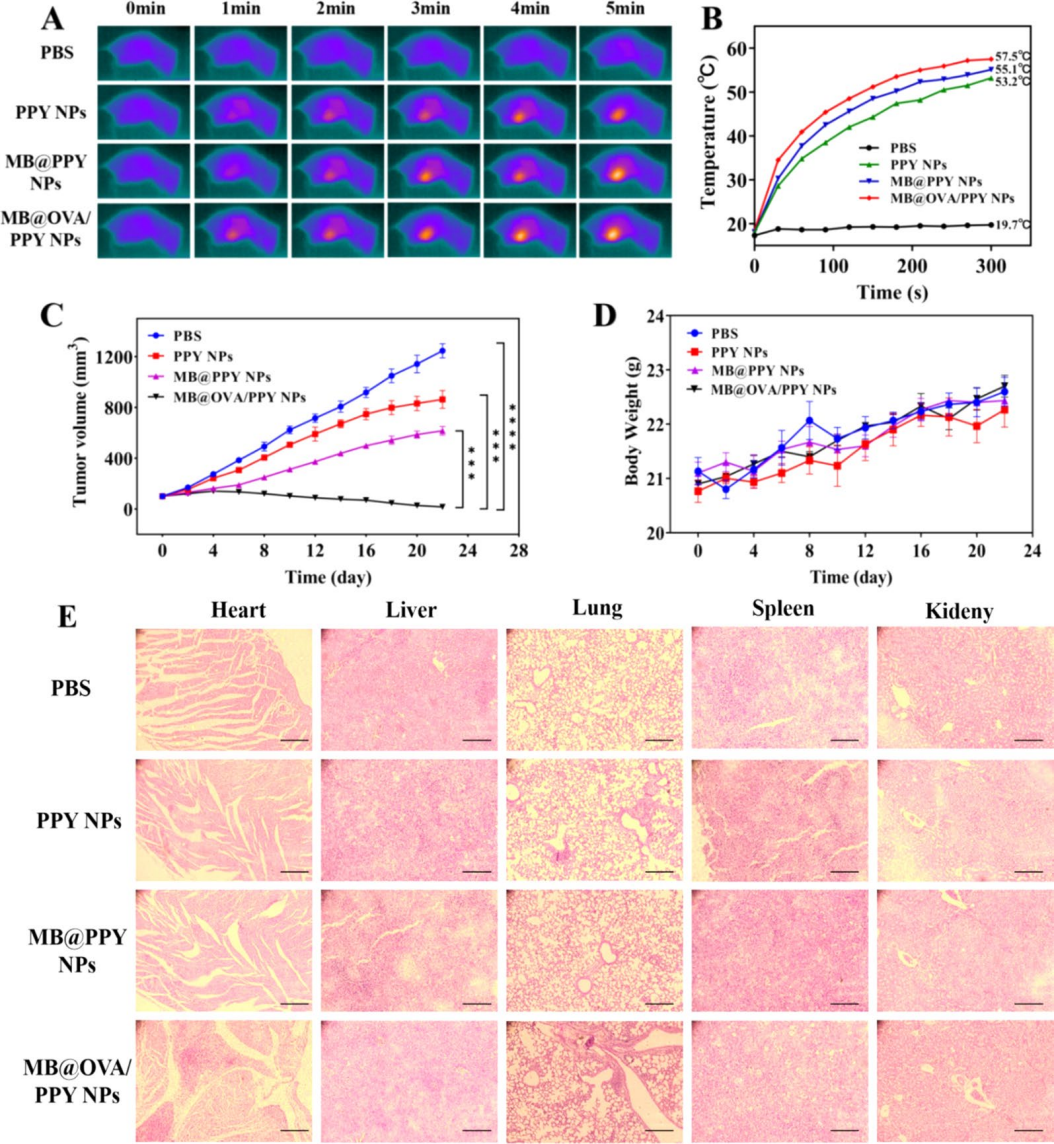
In vivo antitumour effects of MB@OVA/PPY NPs using a 4T1 subcutaneous tumour-bearing mouse model. **A** Infrared thermal images of mice at various time points during treatment under different conditions with 808 nm laser irradiation. **B** Corresponding temperature change curves of the breast tumour sites of the mice in different treatment groups upon laser irradiation. **C** Tumour volume change curves and **D** the body weight changes of the mice in the different treatment groups. Data are expressed as the means ± SD (n = 4) (***p < 0.001, ****p < 0.0001 by t test). **E** H&E-stained images of the major organs in the different treatment groups. Scale bar: 100 µm

To evaluate the photothermal performance in vivo, the changes in temperature at the tumour site were measured after in situ injection of MB@OVA/PPY NPs and 808 nm laser irradiation; additionally, and the photodynamic performance was measured under 660 nm laser irradiation. As shown in Fig. 8A, the temperatures at the tumour sites were significantly increased over time in all groups except the PBS group, which was ascribed to the introduction of polypyrrole nanoparticles. Notably, the tumour temperature in the MB@OVA/PPY NP group reached the highest value (up to 57.5 °C) after laser irradiation for 5 min, as shown in Fig. 8B, but the temperature of the tumours in the PBS group did not noticeably change (reaching only 19.7 °C). Afterwards, irradiation was performed every other day, repeated 3 times, over 22 days, and the corresponding tumour volumes were recorded at certain time intervals. In solid tumours, PTT as a physical method is almost impossible to kill all tumour cells due to the irregularity of tumour tissue. To remedy this issue, we introduced photodynamic therapy on the basis of photothermal therapy. Different from photothermal therapy, photodynamic therapy relies on irradiation with a specific wavelength of light to activate photosensitizers in the tumour tissue to produce reactive oxygen species such as biotoxic singlet oxygen (reactive oxygen species, ROS), which in turn oxidatively damages tumours and can activate antitumour immunity. PDT, as a chemical method, possesses ideal performance to attack these residual tumour cells due to its flexibility. As shown in Fig. 8C, compared with the PBS group, under photothermal action after 808 nm laser irradiation, the tumour volume decreased significantly. Additionally, due to the combined photothermal and photodynamic effects, the MB/PPY NP group showed stronger antitumour consequences than the PPY NP group under dual laser irradiation at 808 nm and 660 nm. The increase in tumour volume in the MB@OVA/PPY NP group was significantly suppressed compared with the other groups, and the tumours disappeared on Day 8. These results demonstrated that MB@OVA/PPY NPs had outstanding antitumour efficacy under dual laser irradiation and immune activation, and the immunological effects were greatly improved. Thus, the combination of laser therapy and the antitumour immune response resulted improved tumour suppression [32, 33]. In addition, there was no significant change in body weights in the mice in all groups, as shown in Fig. 8D. Finally, we further explored the potential risks of the MB@OVA/PPY NPs to major organs in vivo. After treatment, the hearts, livers, spleens, lungs and kidneys of the mice were harvested for H&E staining analysis. The images in Fig. 8E show that there were no inflammatory lesions or obvious tissue damage.

## Conclusions

In summary, we developed a PYY NPs-based nanoplatform to integrate the immune agonist (OVA) and the photosensitizer (MB) for combination therapy. The multifunctional MB@OVA/PPY NPs were successfully synthesized via the iron ion-mediated oxidation polymerization and π–π stacking interactions. The prepared MB@OVA/PPY NPs with a uniform size distribution exhibited favourable water dispersibility and biocompatibility. Under 808 nm NIR irradiation, the photothermal conversion efficiency reached 38%. Additionally, MB@OVA/PPY NPs could generate large amounts of ROS under 660 nm laser irradiation. The combination of PTT/PDT (physical therapy and chemotherapy) showed significant effects on killing tumour cells. The combination of PDT and PTT mediated by MB@OVA/PPY NPs significantly suppressed 4T1 cell growth in vitro by inducing the immunogenic death of tumour cells. More importantly, MB@OVA/PPY NPs significantly promoted the maturation and antigen presentation capability of DCs and elicited a strong and persistent antitumour immune response in vivo. These MB@OVA/PPY NPs have the potential to serve as a novel nanoplatform to synergistically combine PDT/PTT with immunotherapy, which provides a new idea for tumour therapy.

## Data Availability

The datasets supporting the results of this article are included within the article.
